# Chemical characterization, antioxidant and antimicrobial activity of propolis obtained from *Melipona quadrifasciata quadrifasciata* and *Tetragonisca angustula* stingless bees

**DOI:** 10.1590/1414-431X20187118

**Published:** 2018-05-21

**Authors:** A.R. Torres, L.P. Sandjo, M.T. Friedemann, M.M. Tomazzoli, M. Maraschin, C.F. Mello, A.R.S. Santos

**Affiliations:** 1 Programa de Pós-graduação em Farmacologia, Centro de Ciências da Saúde, Universidade Federal de Santa Maria, Santa Maria, RS, Brasil; 2 Laboratorio de Neurobiologia da Dor e Inflamação, Departamento de Ciências Fisiológicas, Centro de Ciências Biológicas, Universidade Federal de Santa Catarina, Florianópolis, SC, Brasil; 3 Departamento de Ciências Farmacêuticas, Centro de Ciências da Saúde, Universidade Federal de Santa Catarina, Florianópolis, SC, Brasil; 4 Laboratório de Morfogênese e Bioquímica Vegetal, Centro de Ciências Agrárias, Universidade Federal de Santa Catarina, Florianópolis, SC, Brasil

**Keywords:** Stingless bees, Propolis, Antimicrobial activity, S. aureus, M. quadrifasciata quadrifasciata, T. angustula

## Abstract

In this study, we investigated the chemical composition, and antioxidant and antibacterial properties of ethanolic extracts of propolis (EEP) from *Melipona quadrifasciata quadrifasciata* and *Tetragonisca angustula*. Chemical composition of EEP was determined by colorimetry and chromatographic (HPLC-DAD and UPLC-Q/TOF-MS/MS) analysis. Antimicrobial activity of EEP was evaluated against gram-positive (*S. aureus*, methicillin-resistant *S. aureus*, *E. faecalis*) and gram-negative (*E. coli* and *K. pneumoniae*) bacteria by the minimal inhibitory concentration (MIC) test using the microdilution method. Furthermore, the growth curve and integrity of cell membrane of *S. aureus* and *E. coli* were investigated using standard microbiological methods. HPLC-DAD analysis showed that the EEP of *M. quadrifasciata quadrifasciata* has a more complex chemical composition than the EEP of *T. angustula*. Moreover, UPLC-MS analyses of *M. quadrifasciata quadrifascita* indicated flavonoids and terpenes as major constituents. The bactericidal activity of both EEPs was higher against gram-positive bacteria than for gram-negative bacteria. The EEP from *M. quadrifasciata quadrifasciata* presented MIC values lower than the EEP from *T. angustula* for all tested bacteria. The EEP from *M. quadrifasciata quadrifasciata* caused lysis of the bacterial wall and release of intracellular components from both *E. coli* and *S. aureus*. Our findings indicate that the chemical composition of propolis from stingless bees is complex and depends on the species. The extract from *M. quadrifasciata quadrifascita* was more effective against gram-positive than gram-negative strains, especially against *S. aureus* and methicillin-resistant *S. aureus* compared to *T. angustula* extract, by a mechanism that involves disturbance of the bacterial cell membrane integrity.

## Introduction

Propolis is a complex mixture of pollen and resinous and balsamic substances collected by bees from buds, flowers, and plant exudates, and bee salivary secretions (1). Since propolis is a bee product of plant origin, its chemical composition and biological activity depends on the specificity of the local flora, season of harvest, and bee species (2–4).

Different biological and therapeutic properties have been reported for propolis, including antioxidant (3,5), anti-inflammatory (5,6), immunomodulatory (7,8), antitumoral (8,9), and antimicrobial activities (2,7,10,11) among others. It has been shown that propolis has bactericidal and bacteriostatic activity against various gram-positive bacteria, such as *S. aureus*, *S. mutans* and *B. subtilis*, and gram-negative bacteria, including *E. coli*, *K. pneumoniae* and *P. aeruginosa* (6,7,9,10). Moreover, a synergistic inhibitory effect of propolis and antibiotics on the growth of *S. aureus* has been reported (11). Such an antimicrobial activity of propolis is particularly relevant if one considers the increasing emergence of antibiotic-resistant microorganisms in hospitals and in the community (11). This situation is aggravated by the inadequate use and prescription of antibiotics and the scarcity of new drugs (12).

Most of the studies in the literature have investigated the antimicrobial activity of the propolis produced by *Apis mellifera*. However, little is known about the biological effects of the propolis produced by other bees, such as the Meliponines. *Melipona quadrifasciata quadrifasciata* Lepeletier and *Tetragonisca angustula* Letreille stingless bees belong to the Meliponini tribe, and are two among more than 200 species of Brazilian native stingless bees (13). Native from tropical and subtropical regions, *M. quadrifasciata quadrifasciata* and *Tetragonisca angustula* are locally known as Mandaçaia and Jataí, respectively. Interestingly, the propolis from *M. quadrifasciata quadrifasciata* is known as geopropolis because it presents soil traces in its composition (14). Due to the unique behavioral and morphological characteristics of these bees, one might reasonably hypothesize that the propolis produced by them has distinct composition and biological activity. Thus, the aim of this work was to characterize the chemical composition of the ethanolic crude extract of propolis (EEP) produced by *M. quadrifasciata quadrifasciata* and *T. angustula* and investigate its potential antioxidant and antibacterial activity against gram-negative and gram-positive bacteria, including methicillin-resistant *S. aureus*.

## Material and Methods

### Chemicals and reagents

DPPH (2,2-diphenyl-1-picrylhydrazyl), resazurin, Folin-Ciocalteu phenol reagent (2N), gallic acid monohydrate (C_7_H_6_O_5_.H_2_O), quercetin, aluminum chloride (AlCl_3_) and sodium carbonate (Na_2_CO_3_) were purchased from Sigma (USA). Ethanol and methanol were obtained from Merck (Brazil). Acetonitrile was from Tedia (Brazil). The culture medium Brain Heart Infusion was obtained from Himedia (India). The bacteria strains were obtained from Laborclin (Brazil) and Microbiology Laboratory of the Federal University of Santa Maria (Brazil). All other chemicals were of analytical grade and purity. Aqueous solutions were prepared in ultrapure water produced by a Milli-Q system (18.2 MΩ, Millipore, France).

### Propolis samples and ethanolic extract preparation

The samples were collected in September of 2014 in Rio das Antas, Brazil. Five samples of the propolis from *M. quadrifasciata quadrifasciata* and three samples from *T. angustula* were obtained from the inner parts of the beehives. The ethanolic extract was prepared as reported by Park et al. (15). Two grams of the powder was mixed with 25 mL of 80% ethanol in a sealed container protected from light (to avoid loss of volatile and photosensitive compounds), under agitation in a water bath at 70°C for 30 min. After extraction, the mixture was filtered (grade 1 Whatman) to obtain the EEP at concentration of 80 mg/mL (propolis:ethanol 80%, w/v).

### Total polyphenol and flavonoid contents

The total polyphenol content of EEP was determined using the Folin-Ciocalteu colorimetric method described by Frozza et al. (16). Briefly, 100 µL of the hydroalcoholic extract (1 mg/mL) was mixed with 500 µL of Folin-Ciocalteu and after 5 min in dark, 400 µL sodium carbonate (7.5%) was added. After incubation in the dark at room temperature for 30 min, the absorbance of the reaction mixture was measured at 765 nm in a spectrophotometer (model FlexStation, Molecular Devices, USA). Gallic acid standard solutions (0.25–4.0 µL/mL) were used for the calibration curve. The average of three readings was used to determine the total polyphenol content, reported as mg of gallic acid equivalents per g of propolis (GAEs).

The total flavonoid content in EEP was determined by the method described by Campos et al. (9). For this, 0.5 mL of EEP (100 µg/mL) was mixed with 4.5 mL of 2% aluminum chloride hexahydrate in methanol. After 30-min incubation at room temperature in the dark, the absorbance was read at 415 nm using a plate spectrophotometer (FlexStation, Molecular Device). Quercetin (0.4–11 µg/mL) was used as standard. Triplicates were used to determine the flavonoid content, reported as mg of QE per g of propolis.

### High performance liquid chromatography (HPLC-DAD) analysis

Briefly, 10-μL samples of EEP were injected in the liquid chromatographer (Thermo Scientific Dionex UltiMate 3000, USA), equipped with a C18 reverse phase column (BioBasic-18, 150 mm × 4.6 mm Ø, 5 μm) thermostatized at 40°C and diode array detector. Elution occurred with a flow rate of 0.8 mL/min using a linear gradient of a formic acid aqueous solution 0.5% (v/v) (solvent A) and methanol (solvent B) as follows: (0–10 min) 15% B, (10–55 min) gradual increase to 70% B and (55–60 min) gradual reduction to 15% B. The identification of the phenolic compounds was carried out by comparing the retention time of the samples with pinocembrin, quercetin, ρ-coumaric acid, chrysin, gallic acid, and artepillin C standards.

### Ultra performance liquid chromatography (UPLC) analysis/ESI-QTOF-mass spectrometry (MS)

The propolis extract (1 mg/mL) was filtered with a syringe filter (13 mm, 0.22 μm, Analítica, Brazil) before the analysis. Chromatographic separation was carried out in an Acquity UPLC system class H (Waters, USA) equipped with a PDA 9-detector, sample manager, and a quaternary solvent manager as well as a BEH C18 column: 100 mm, 1.0 mm, particle size 1.7 μm (Waters). The temperature of the column and the sample tray were 40°C and 20°C, respectively. The gradient used for the separation (flow rate of 0.3 mL/min) was composed of A [water/formic acid, 99.9/0.1 (v/v)] and B (acetonitrile). The elution was made as follows; 0.0–4.9 min 50% of A; 5–9 min 40% of A; 9.1–12 min 10% of A; 12.1–14.9 min 5% of A; 15–20 min 95% of A. The injection volume was 2 µL.

Mass data were recorded on a Xevo G2-S QTof (Waters) equipped with an electrospray ionization source operating in positive (ESI+) and negative (ESI-) ion modes using the following instrument settings: nebulizer gas: nitrogen; cone gas flow 10 L/h; desolvation gas flow 900 L/h; sampling cone 40 V; source offset 80 V; collision gas: argon; Lockspray reference sample: Leucine enkephalin. Lock masses are m/z 556.2771 (ESI+) and m/z 554.2615 (ESI-).

#### LC infusion (ESI+)

The desolvation and source temperatures were set at 300 and 90°C, respectively. The capillary voltage was set to 3 kV. Data were collected between 100 and 1200 Da, with a scan time of 1.0 sec over an analysis time of 20 min. The LC-MS/MS analyses were performed with a collision energy of 25 eV.

#### LC infusion (ESI-)

The desolvation and source temperatures were set at 300 and 90°C, respectively. The capillary voltage was set to 2.5 kV. Data were collected between 100 and 1200 Da, with a scan time of 1.0 sec over an analysis time of 20 min. The LC-MS/MS analyses were performed with a collision energy of 25 eV. Data was processed with the MassLynx V4.1 software (Waters).

### Antioxidant activity

The DPPH free radical scavenging activity was measured according to Campos et al. (9), with minor modifications. Briefly, 150 µL of various concentrations of EEP were mixed with 150 µL of DPPH stock solution [80 µmol/L in ethanol at 80% (v/v)]. The mixture was incubated at room temperature in the dark for 20 min and absorbance was measured at 517 nm in a plate spectrophotometer (FlexStation, Molecular Devices). Extract concentrations were plotted against respective inhibition of DPPH reduction and IC_50_ was estimated by nonlinear regression using data from three independent experiments carried out in triplicate.

### Determination of minimal inhibitory concentration (MIC)

The MIC of EEP against *S. aureus* (ATCC 25923), methicillin-resistant *S. aureus* (MRSA, clinic isolate), *E. faecalis* (ATCC 29212), *E. coli* (ATCC 25922), and *K. pneumoniae* (ATCC 23883) was determined by the broth microdilution method, which was performed according to the Clinical and Laboratory Standards Institute - CLSI M.07-A.9 (17), with minor modifications. The bacterial strains were inoculated in Brain Heart Infusion (BHI) broth with different concentrations of EEP (16–0.25 mg/mL) in 96-well microplates and incubated at 37°C for 24 h. The bacterial inoculum density was adjusted to 10^8^ CFU/mL according to the 0.5 MacFarland scale and diluted to obtain a final concentration of 5×10^5^ CFU/mL. After 24 h of incubation, 30 µL of resazurin at 0.01% (w/v) was added and after 30 min the samples were visually inspected (18). The color change from purple to pink was recorded as positive bacterial growth. The inoculated medium was used as positive control (growth control), culture medium was used as negative control (sterility control), and a diluent control was made in each experiment. The MIC was considered as the lowest concentration of EEP that inhibited growth. Five independent experiments were performed for each bacterial strain.

### Growth curve

The growth curve assay was used to investigate the bactericidal effects of EEP (0, 0.5, 1, or 2 MIC) over time (0, 2, 4, 6, 8, 12, and 24 h intervals). For this, 100 µL of EEP and 100 µL of bacterial inoculum, both previously diluted in BHI broth. The inoculum was diluted to obtain a final concentration of 5×10^5^ CFU/mL. After each incubation, 10 µL resazurin (0.01%) was added to the withdrawn sample and the mixture was incubated at room temperature in the dark for 5 min. The mixture was then centrifuged at 10,000 *g* for 10 min at room temperature and the absorbance of the supernatant was measured at 550 nm.

### Integrity of cell membrane

The bacterial cell membrane integrity was assessed by measuring the release of cell constituents into supernatant according to Diao et al. (19), with minor modifications. Bacterial cultures (100 mL) were incubated overnight at 37°C and centrifuged at 3500 *g* for 15 min at room temperature, washed three times and resuspended in 0.1 M phosphate buffer solution (PBS, pH 7.4). The cell suspension absorbance was adjusted to 0.5 at 620 nm with PBS. Two hundred microliters of 0.1 M PBS (negative control, 0 MIC) or EEP (1 MIC) were added to 1.8 mL of bacterial suspension. The suspensions were incubated at 37°C for 4 h, with periodic agitation. Samples were then centrifuged at 11,000 *g* for 5 min at 4°C and 200 µL of the supernatant was removed to assess the released content (largely nucleic acids) by measuring absorbance at 260 nm (SpectraMax, Molecular Devices). Absorbance values were corrected using adequate control blanks containing EEP and PBS (pH 7.4).

### Statistical analysis

Data were analyzed by the *t*-test or one-way ANOVA followed by Bonferroni’s test depending on the number of groups. IC_50_ was determined by nonlinear regression. All analyses were performed using GraphPad Prism version 6.07 for Windows, GraphPad Software, USA.

## Results

### Total polyphenol and flavonoid content

The polyphenol content was 3.87±0.32 and 1.26±0.17 mg of GAE/g of propolis for *M. quadrifasciata quadrifasciata* and *T. angustula*, respectively. The flavonoid content was 0.14±0.03 mg QE/g of propolis for *M. quadrifasciata quadrifasciata* and 0.15±0.02 mg QE/g of propolis for *T. angustula*. Only the polyphenol content was significantly different between the EEPs of the two bee species (P<0.001).

### HPLC-DAD analysis

**Figure 1.**
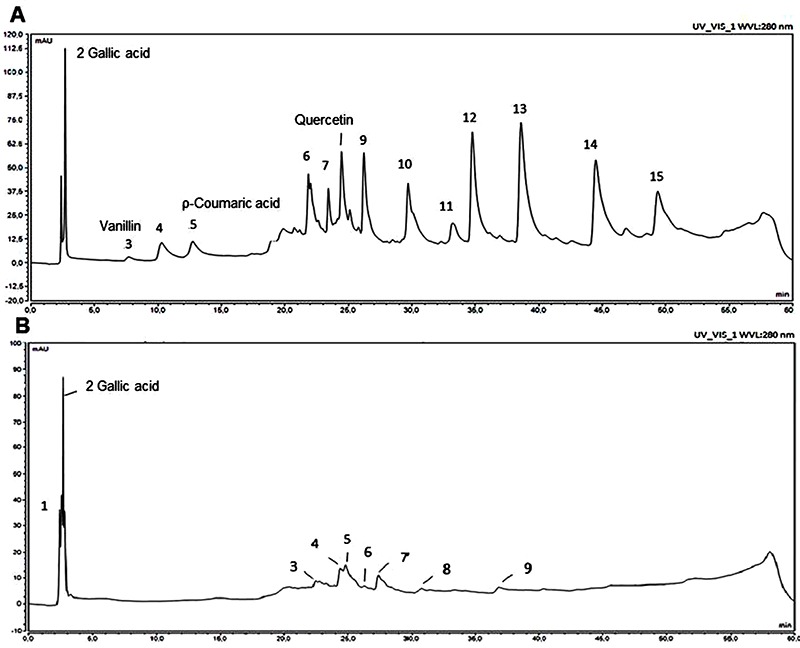
. HPLC-DAD (280 nm) chromatograms of the ethanolic extract of propolis using a C18-reversed phase column at 40°C. *A*, *M. quadrifasciata quadrifasciata* peaks: 2-gallic acid (Rt=2.68 min), 3-vanillin (Rt=7.67 min), 5-ρ-coumaric acid (Rt=12.73 min), and 8-quercetin (Rt=24.45 min). *B*, *T. angustula* peak: 2-gallic acid (Rt=2.69 min). Rt: retention time. The peaks not cited are from unidentified compounds.

HPLC chromatograms are presented in Figure 1A and 1B. The analysis of the propolis from *M. quadrifasciata quadrifasciata* revealed the presence of gallic acid, vanillin, ρ-coumaric acid, and quercetin (retention times: 2.68, 7.67, 12.73, and 24.45 min, respectively). The analysis of the propolis from *T. angustula* revealed the presence of gallic acid (retention time: 2.68 min).

### UPLC analysis/ESI-QTOF-MS

UPLC-ESI-QTOF-MS/MS techniques showed a good separation profile for the EEP from *M. quadrifasciata quadrifasciata*. The analysis in positive and negative ionization modes revealed the presence of 26 diterpenes skeletons as major components, of which 17 were characterized (Table 1). The identification was supported by data found in the literature, based on which some of these propolis constituents were found to be sesquiterpene metabolites, triterpenes, stilbenes, and polyphenols.

**Table 1.** Identification of compounds in ethanolic crude extract from the propolis of *M. quadrifasciata quadrifasciata* by UPLC-MS/MS and ESI-QTOF/MS analysis, in negative and positive mode.

t_R_ (min)	Mol. weight (m/z)	Calc. mass (m/z)	Elem. comp.	Fragments (m/z)	Proposed structure	Ref.
**ESI- ([M-H]^-^)**						
4.71	291.1586	291.1596	C_17_H_24_O_4_	273.1490, 245.1571, 229.1601, 213.1283	pinusenocarp	35
5.34	335.2220	335.2222	C_20_H_32_O_4_	317.2103, 299.1998	junicedric acid or salvicin	36
5.48	331.1913	331.1909	C_20_H_28_O_4_	313.1810, 269.1823, 255.1380, 227.1429	inumakiol D	37
6.95	319.2274	319.2273	C_20_H_32_O_3_	300.2069, 231.1707	isocupressic acid	20
7.36	317.2103	317.2117	C_20_H_30_O_3_	299.1998, 271.1856, 221.1538	agathalic acid	20
7.50	317.2103	317.2117	C_20_H_30_O_3_	299.1998, 287.1987, 273.2234, 271.2092, 257.1837, 255.2166, 253.2015	15-oxolabda-8(17), 13Z-diene-19-oic acid or (15-oxolabda-(17),13E-dien-19-oic acid) or agathalic acid	20, 36
9.45	347.2197	347.2222	C_21_H_32_O_4_	-	15-agathic acid methyl ester	20
11.28	301.2157	301.2171	C_20_H_30_O_2_	-	trans-communic acid or pimaric acid	36
**ESI+ ([M+H]^+^)**						
6.95	303.2305	303.2324	C_20_H_30_O_2_	285.2144, 267.2139, 257.2272, 215.1833, 201.1677	hinokiol, or 3β-hydroxytotarol or totara-8,11,13-triene-7α,13-diol	21, 22
7.36	301.2183	301.2168	C_20_H_28_O_2_	283.2144, 255.2140, 199.1512, 185.1365, 173.1339	angustanoic acid A	38
7.50	301.2183	301.2168	C_20_H_28_O_2_	283.2144, 255.2140, 199.1512, 185.1365, 173.1339	Related to angustanoic acid A	-
7.91	315.1970	315.1960	C_20_H_26_O_3_	271.2100, 227.1460, 213.1305, 199.1137, 187.1145, 175.1145, 171.0843, 149.0993	artepillin C methyl ether	-
8.09	327.1584	327.1596	C_20_H_22_O_4_	-	(E)-4-(3-methyl-2-buten-1-yl)-3,3′,5-trihydroxy-4′-methoxystilbene or (E)-2-(3-methyl-2-buten-1-yl)-3′,4′,5-trihydroxy-3-methoxystilbene	39
8.35	327.1584	327.1596	C_20_H_22_O_4_	-	related to (E)-4-(3-methyl-2-buten-1-yl)-3,3′,5-trihydroxy-4′-methoxystilbene	39
8.53	287.2378	287.2375	C_20_H_30_O	-	trans-totarol or trans-communal	36
11.83	303.2305	303.2324	C_20_H_30_O_2_	257.2305, 255.2142, 201.1677, 187.1508, 173.1366, 149.1343, 135.1183, 123.1199	related to *trans*-communic acid or pimaric acid	36
13.96	441.3735	441.3733	C_30_H_48_O_2_	-	24(E)-3β-hydroxycycloart-24-ene-26-al	40

The major component detected at m/z 319.2274 [C_20_H_32_O_3_-H]^-^ (t_*R*_ 6.95 min) in the negative ionization mode was identified as isocupressic acid (20). Only two fragments were obtained from this precursor (m/z 231.1707 and 300.2069). The fragment m/z 300.2069 corresponded to loss of the neutral species H_2_ and the OH radical while the other was found after considering the decarboxylation (loss of CO_2_), the elimination of CH_4_ and C_2_H_4_ resulting in the opening of the left ring of the decalin portion.

The structure of hinokiol, 3β-hydroxytotarol, or totara-8,11,13-triene-7α,13-diol (21,22) was proposed for the major compound detected in the positive mode at 6.95 min (m/z 303.2305: [C_20_H_30_O_2_+H]^+^). In fact, all three compounds could successively loose two molecules of H_2_O to generate m/z 285.2242 [M+H-H_2_O]^+^ and m/z 267.2139 [M+H-2H_2_O]^+^, respectively. Their phenol could also isomerize to a ketone and a ring constriction could occur by elimination of carbon monoxide to give m/z 255.2142 [M+H-H_2_O-CO]^+^. Furthermore, an isopropylene moiety could also be eliminated from the precursor m/z 255.2142 yielding m/z 215.1833 [M+H-H_2_O-CO-C_3_H_6_]^+^.

### Antioxidant activity

**Figure 2.**
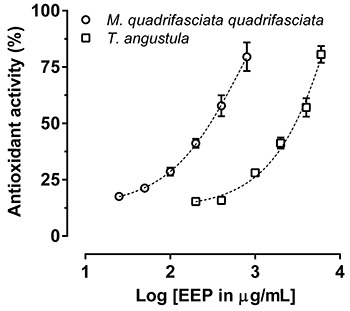
Effect of ethanolic extracts of propolis (EEP) from *M. quadrifasciata quadrifasciata* and *T. angustula* on DPPH radical scavenging. IC_50_ values were obtained by nonlinear regression; M. *quadrifasciata quadrifasciata*: IC_50_=241.8 µg/mL and *T. angustula*: IC_50_=2433.0 µg/mL. Data are reported as means±SE of three independent experiments performed in triplicate.

The results reported in Figure 2 show that both EEPs had dose-dependent antioxidant activity. Moreover, the EEP from *M. quadrifasciata quadrifasciata* [IC_50_= 241.8 (203.1 to 287.7) µg/mL] was ten-fold more potent than the EEP from *T. angustula* [IC_50_= 2433.0 (2086.0 to 2838.0) µg/mL)].

### Determination of MIC

**Figure 3.**
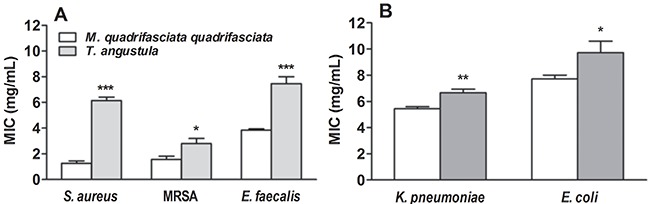
Susceptibility of bacterial strains to ethanolic extracts of propolis in minimal inhibitory concentration (MIC). *A*, Gram-positive bacteria: *S. aureus* (ATCC 25923), methicillin-resistant *S. aureus* (MRSA, clinical isolate), and *E. faecalis* (ATCC 29212); *B*, Gram-negative bacteria: *K. pneumoniae* (ATCC 23883) and *E. coli* (ATCC 25922). Data are reported as means±SE of 3–5 independent experiments performed in triplicate. *P<0.05, **P<0.01 and ***P<0.001 compared to *M. quadrifasciata quadrifasciata* (ANOVA followed by Bonferroni’s test).

The MIC values of the extracts for gram-positive and gram-negative bacteria are shown in Figure 3A and 3B. Gram-positive bacteria (*E. faecalis*, *S. aureus* and MRSA) were more sensitive than gram-negative bacteria (*E. coli* and *K. pneumoniae*) to both EEPs. In addition, the EEP from *M. quadrifasciata quadrifasciata* was more potent and efficacious than the EEP of *T. angustula* showing the lowest MIC values for all tested bacteria.

### Growth curve

**Figure 4.**
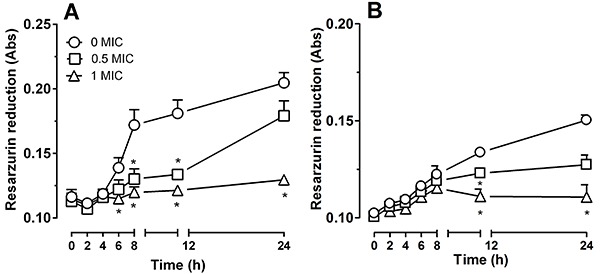
Effect of ethanolic extracts of propolis from *M. quadrifasciata quadrifasciata* on growth curve assay. Absorbance readings for assays with *S. aureus* (*A*) and *E. coli* (*B*). Data are reported as means±SE of three independent experiments performed in triplicate. *P<0.05 compared to respective control (*t*-test).

Considering the promising results in the MIC assay, we decided to investigate the effect of the EEP from *M. quadrifasciata quadrifasciata* on the growth of *S. aureus* (ATCC 25923) and *E. coli* (ATCC 25922) over time. Figure 4 shows that the inhibitory effect of *M. quadrifasciata quadrifasciata* (1 MIC) EEP on *S. aureus* growth was time-dependent and occurred in about 6 h. On the other hand, the inhibitory effect of the extract on the growth of *E. coli* took 12 h to occur.

### Integrity of cell membrane

The results presented in Table 2 show that the EEP from *M. quadrifasciata quadrifasciata* (1 MIC) increased 6.6- and 5.6-fold the leakage of cell constituents of *S. aureus* and *E. coli*, respectively, suggesting that it causes an irreversible damage of the bacterial cell membrane, leading to cell death.

**Table 2.** Effect of ethanolic extracts of propolis (EEP) from *M. quadrifasciata quadrifasciata* on cell constituents release of *S. aureus* and *E. coli* after 4 h.

Bacterial Strains	Cell Constituents Release (OD_260 nm_)		Relative Release (EEP/Control)
	Control	EEP (1 x MIC)	
*S. aureus*	0.041±0.004	0.260±0.033*	6.61±1.211
*E. coli*	0.149±0.017	0.811±0.054**	5.65±0.585

Data are reported as means±SE of three independent experiments performed in triplicate. *P=0.0004 and **P=0.0001 compared to respective control (*t*-test).

## Discussion

The current study revealed that EEPs from *M. quadrifasciata quadrifasciata* and *T. angustula* had antimicrobial activity against gram-positive and gram-negative bacteria and antioxidant activity. The chemical analysis of the EEPs revealed the presence of terpenoids, flavonoids, and polyphenols, which were more abundant in the EEP from *M. quadrifasciata quadrifasciata* (Figure 1).

The more prominent effect of EEPs against gram-positive than against gram-negative bacteria, as assessed by the MIC assay, agrees with previous studies that have shown that propolis from stingless bees (7,9,23) and from *Apis mellifera* (6) has antimicrobial activity, particularly against gram-positive bacteria. In this regard, the currently reported activity against MRSA is particularly interesting due to the present scenario of recrudescence of resistant *S. aureus* strains (12). The MICs estimated in the current study for gram-positive bacteria are similar to the estimated MICs of EEPs from other stingless bees, around 2–3 mg/mL for *S. aureus* (9,23), including MRSA. Previous studies that have used the same experimental protocol of MIC determination used in our study could not determine a MIC for EEP against gram-negative bacteria (9). However, we found MIC values for EEP against gram-negative bacteria between 5 and 7 mg/mL in our samples, also indicating some activity of EEP from *M. quadrifasciata quadrifasciata* and *T. angustula* against gram-negative bacteria. Considering the estimated MICs in our assays, the EEP from *M. quadrifasciata quadrifasciata* was more potent than the EEP from *T. angustula* as an antimicrobial agent. Although the EEPs showed important antimicrobial activity for all tested strains, MICs values obtained (2 to 7 mg/mL) can be considered high, conferring a reasonable antimicrobial activity.

Due to its better antimicrobial activity, the EEP from *M. quadrifasciata quadrifasciata* was chosen for additional tests: growth curve, release of cell constituents, and mass spectrometry experiments. To analyze the effect of the EEP from *M. quadrifasciata quadrifasciata* against *S. aureus* and *E. coli* over time, a growth curve assay was performed in the absence or presence of the EEP (1 MIC). *S. aureus* was more susceptible to EEP than *E. coli* also in this assay. Accordingly, while a significant growth reduction was found at 6 hours for *S. aureus*, 12 hours were necessary to show a significant growth reduction for *E. coli*, compared to their respective controls (0 MIC).

Although some authors attribute the bacteriostatic and bactericidal activity of propolis to the inhibition of protein synthesis and prevention of cell division (24), its nature and complexity complicate the identification of a mechanism of action. In this study, we performed a cell constituent release assay to investigate a possible mechanism of action for EEP, i.e. disruption of the cell membrane, which would cause the release of large molecules to the medium. The assay revealed a significant release of intracellular constituents of *S. aureus* and *E. coli* to the incubation medium in the presence of the EEP from *M. quadrifasciata quadrifasciata* (Table 2), supporting that it causes cell lysis.

Aiming to further elucidate the composition of EEP from *M. quadrifasciata quadrifasciata*, an UPLC coupled with mass spectrometry assay was carried out. The assay showed 26 diterpene skeletons as major components and, based on the literature, it was possible to suggest 17 structures. Among these, the following compounds are particularly relevant: one of elemental composition C_20_H_30_O_2_, which may be a hinokiol or totarol derivative, isocupressic acid, and artepillin C methyl ester. The presence of totarol and possibly a derivative is consistent with our antibacterial findings. Totarol is a highly hydrophobic diterpenoid with a high phospholipid/water partition coefficient, capable of interfering with the structural integrity of the membrane of bacteria and causing cell lysis (25). In addition, it decreases the expression of penicillin binding protein 2a, a protein involved in penicillin resistance of MRSA (26). Recent evidence supports that totarol inhibits hemolytic proteins and enterotoxins secreted by *S. aureus* (27) and has potential application in clinical therapy and food decay prevention. In line with this view, hinokiol, also an identified component of EEP from *M. quadrifasciata quadrifasciata*, has been described as having antimicrobial, antitumoral, antioxidant and anti-inflammatory activity (28,29). Therefore, hinokiol may also be involved in the antimicrobial action of EEP from *M. quadrifasciata quadrifasciata*. In addition, isocupressic acid, also a component of propolis, has antimicrobial activity (30 [Bibr B31] [Bibr B32]) and may play a role in the antibiotic effect of EEP from *M. quadrifasciata quadrifasciata.* The UPLC-MS also revealed the presence of artepillin C in the EEP from *M. quadrifasciata quadrifasciata.* Artepillin C has been pointed out as the possible active component responsible for the antimicrobial and antioxidant activity of green propolis (31), similarly to totarol, interacting with cell membrane and creating point defects in its structure (32). Therefore, one might consider that artepillin C is involved in the current antimicrobial effect of EEP from *M. quadrifasciata quadrifasciata.*

It is well known that propolis from different bee species contain significant amount of antioxidants (5). Therefore, we decided to comparatively assess the antioxidant activity and total content of phenols and flavonoids in the EEPs from *M. quadrifasciata quadrifasciata* and *T. angustula.* The EEP from *M. quadrifasciata quadrifasciata* presented higher antioxidant activity than the EEP form *T. angustula* in the DPPH assay (IC_50_=241.8 and 2433.0 µg/mL, respectively). Interestingly, Bonamigo et al. (33) also demonstrated that ethanol extracts of propolis obtained from the stingless bees *M. quadrifasciata anthidioides* had a higher antioxidant capacity in the DPPH (IC_50_=60.9 µg/mL) and ABTS (IC_50_=13.4 µg/mL) assay compared to *Scaptotrigona depilis*. Considering the antioxidant profile of the propolis extract obtained from the *M. quadrifasciata anthidioides* and *M. quadrifasciata quadrifasciata* in the DPPH test, we can observe that the *M. quadrifasciata anthidioides* was about 3.9-fold more potent than the *M. quadrifasciata quadrifasciata*. Based on the above results, we can also suggest that the antioxidant activity present in propolis seemed to depend on the genus and species of bees, considering that the potency and efficacy of the propolis obtained from the bees belonging to the Melipona genus (*M. quadrifasciata anthidioides* and *M. quadrifasciata quadrifasciata*) were higher than Tetragonisca (*T. angustula*) and Scaptotrigona (*S. depilis*), respectively.

The differences in the chemical composition of propolis extracts in the same region may be related to species of bees and the preference for a particular plant species to elaborate the propolis (2,33). Moreover, the genetic variability of bee species influences the chemical composition of propolis, resulting in different biological activities (2). Accordingly, the EEP from *M. quadrifasciata quadrifasciata* presented a higher concentration of total phenols and flavonoids, reinforcing the direct correlation between phenol concentration and antioxidant activity established in the literature (34 [Bibr B35] [Bibr B36] [Bibr B37] [Bibr B38] [Bibr B39] [Bibr B40]).

In conclusion, the data presented here showed that the chemical composition of propolis from stingless bees is complex and depends on the species, among other factors. The extract from *M. quadrifasciata quadrifascita* was more potent in promoting antioxidant and antibacterial activity compared to *T. angustula* extract. In addition, EEPs were more effective against gram-positive than against gram-negative strains, especially against *S. aureus* and MRSA, by a mechanism that involved the disturbance of bacterial cell membrane integrity. The current findings suggest that propolis from stingless bees may be a potential source of active compounds against MRSA.
